# Genito-Urinary and Syphilitic Diseases

**Published:** 1897-11

**Authors:** Byron H. Daggett

**Affiliations:** Buffalo, N. Y.


### Progress in Medical Science.

### GENITO-URINARY AND SYPHILITIC DISEASES.

Conducted by BYRON H. DAGGETT, M. D., Buffalo, N. Y.

### ELIGIBILITY OF GONORRHEIC APPLICANTS FOR LIFE INSURANCE.

DR. BUTTERWORTH (*Medical Examiner*, November, 1896,) says that this subject is deeply interesting to the medical mind, the insurance company and the majority of mankind, when it is remembered that a large percentage of applicants for life insurance, especially those living in towns and cities, have, at some time during their life, made the acquaintance of this undesirable malady, the immediate results of which are often disastrous to health ; the remote results being manifested years after infection often cause great physical suffering and not infrequently death.

A recent authority states that he has found 15 per cent. of the cases coming under his observation suffering from cardiac lesions, following gonorrheal arthritis. Undoubtedly the excessive use of drugs by chronic gonorrheics often impairs the integrity of the kidneys and the tendency of this disease in a latent form is to recrudescence from excesses ; thus it becomes a menace to the associated urinary and genital organs, in its acute as well as in its chronic stage, and spreads by continuity of tissue and involves the remotest organs of these systems.

Gonorrhea is more rebellious to treatment and is a more fatal malady than syphilis. An eminent authority alleges that 90 per cent. of these cases are never cured. Surely numerous cases drag along many years without heed from its victims and unrecognised by their physicians. The continuous use of salol and the injudicious use of oleo-resins and balsams cause albuminuria and kidney irritation. Many pathologic sequelæ result from gonorrhea and its improper treatment. While the results of gonorrhea in the female are often very grave, they are slow and insidious in their progress. Owing to the different anatomical formation of the female genito-urinary system, woman suffers more from infection of the genitalia, while the male suffers more from disturbance of the urinary system. Gonorrhea and its results are frequently responsible for deaths attributed to other causes. Dr. Butterworth states that this disease and its bearings on life insurance have received insufficient attention from medical examiners.

Individual genital and venereal history is regarded as a closed chapter, a "*noli me tangere.*" For these reasons this subject does not receive the attention that its importance demands at the hands of the medical examiner and the insurance company.

Gonorrhea at any stage impairs the grade of the risk ; it renders its victim vulnerable and often becomes a veritable Herod to the unfortunate female who receives the embraces of the gonorrheic leper.

If, during the examination, any signs of venereal disease are developed, the judicious, tactful physician may elicit the facts without offense and receive the grateful, appreciative approbation of both the applicant and the company for his careful diagnosis.

---

### THE VALUE OF THE THERMO-MINERAL CURE IN THE TREATMENT OF SYPHILIS.

CORLETT (*Cleveland Journal*, December, 1896,) says : This method of treatment hastens elimination, destroys or lessens the virulence of the virus and inhibits or promotes the healing of existing lesions. This holds good in the various forms and phases of the disease and the sooner it is applied the better.

The thermo-mineral cure is especially applicable to the syphilitics dwelling in large cities, in whom mercury and the iodin compounds have failed and in cases of long standing. Fresh air, sunlight, exercise and drinking hot water are equally important with bathing. As this system hastens the elimination of the virus, there is less probability of the late manifestation and development of lesions of the nervous system and there are no counter-indications to its judicious employment.

The mixed thermal and mercurial treatment, in addition to the advantages mentioned, stimulates the eliminative organs, so that a greater quantity of mercury and iodin compounds may be administered without producing their disagreeable effects. Mercury is efficient in destroying the syphilitic virus and causes the disappearance of the earlier lesions of the disease, while the iodin compounds, stimulating the absorbents, heal the later manifestations, without especially preventing their return. During the thermal cure the patient may be mercurialised by any of the usual methods of administration and the mercurialisation should not exceed a slight tenderness of the gums. The dosage varies according to the nature of the case, the age of the disease and the severity of the preceding symptoms. Thermal treatment confers more or less immunity against hydrargyrism.

Dr. Corlett advises the use of plain water, carbonised water and in some cases sulphur water and says that high thermality favors elimination. Plain water daily and a Turkish bath once a week is the form recommended for thermal treatment. Treatment should continue from one to four years.

The author has visited many of the springs in Europe and America. He believes that the vast majority of patients do not give a sufficient time at the springs for the best results ; that one or two months each season, while it relieves present symptoms, is far too short for the permanency of cure.

Most patients visiting these springs content themselves with the belief that the disease is "boiled" out of them and attribute too much importance to the "cure" properties of the waters, thereby neglecting the essentials of successful treatment—namely, the use of mercury and iodin.

---

### CLINICAL CLASSIFICATION OF NEPHRITIS.

Brouet, of Paris, (*Medical Record*, September 11, 1897,) says that there never has been anything like a satisfactory classification of nephritis until the present day, when the pathology of the kidney has been completely changed by the studies and discoveries in pathological histology.

A classification to be of use must be rational and practical, whether it be founded upon clinical or histological data. The histological classification divides nephritis into parenchymatous (Virchow, 1852,) and interstitial (Beer, 1859). This division is accepted by almost all microscopists.

Bartholow recognised an acute and chronic parenchymatous nephritis and an interstitial nephritis or induration of the cellular tissue of the kidneys (atrophy, cirrhosis, sclerosis and the like). Tancereaux divided nephritis into two divisions—namely, primary diffuse and epithelial. Charcot called attention to the identity of parenchymatous nephritis and large, white kidney, and of interstitial nephritis and small, contracted kidney. Neigert, Cohnheim and Wagner said that the epithelial lesions always precede the alteration in the connective tissues and that if the lesions varied in different cases it was due to the difference in the degree of the progress of the disease. Brouet says that the natural classification is an etiological one and contends that there is no longer a question of the unity or duality of Bright's disease.

The size, consistency, form, coloration, granulation and the like, of diseased kidneys, depends on whether the disease process is rapid or slow, violent or mild.

The histological condition rather indicates the degree and progress of the malady than the division or classification of the disease. "Infectious diseases irritate, inflame, destroy *en masse;* chronic diseases, the dyscrasias and prolonged intoxications destroy the organ by a series of local inflammations or cause it to degenerate little by little."

A clinical classification is based upon the duration of the disease ; as transitory, acute, subacute and chronic nephritis, comprehending the latent varieties.

In all cases of prolonged nephritis we have hypertrophy of the heart. Given the cause of the nephritis and the date of its appearance and we have sufficient data to explain what has already taken place ; to prognosticate and to infer the existing anatomical form.

Dr. Crocq, of Brussels, said that albuminuria is the result of desquamation of the renal epithelium, and that we could not have a so-called physiological albuminuria, even as a transitory condition, without this cause. The albuminuria, if of short duration, the cause escapes one's notice. Parenchymatous inflammation of the kidney is usually the cause of the excretion of albumin, which almost invariably points to chronic Bright's disease. Interstitial nephritis is quite another thing. The interstitial form is an inflammation of the connective tissue, which binds together the parenchymatous elements. If the epithelium is not involved there is no excretion of albumin, but albumin appears as soon as the epithelium is affected. There are two forms, clinically speaking, of chronic nephritis ; one with and one without albumin, and these are identical with the anatomical forms called parenchymatous and interstitial.

Dr. Senator, of Berlin, said that when chronic nephritis is not the result of frank, acute nephritis, it comes from latent inflammations of the kidney long continued. The smallest amount of albuminuria is a sign of a pathological process in the kidney. Albumin cannot leak through the kidney except there be an organic affection of that organ. Chronic nephritis is often due to this latent process that so easily escapes notice.

Dr. Gerhardt, of Berlin, did not think that albuminuria was always due to organic lesion of the kidney. In some people there appeared to be a physiological albuminuria.

